# Comprehensive Assessment of Harvesting Method Effects on FAEE, Waxes, Fatty Acids, Phenolics, Volatiles, and Sensory Characteristics of Buža Virgin Olive Oil

**DOI:** 10.3390/foods15010160

**Published:** 2026-01-03

**Authors:** Karolina Brkić Bubola, Marina Lukić, Iva Pastor, Igor Lukić, Gašper Kozlovič, Milena Bučar-Miklavčič, Olivera Koprivnjak, Marin Krapac

**Affiliations:** 1 Institute of Agriculture and Tourism, Karla Huguesa 8, HR-52440 Poreč, Croatia; marina@iptpo.hr (M.L.); ivap@iptpo.hr (I.P.); igor@iptpo.hr (I.L.); marin@iptpo.hr (M.K.); 2 Science and Research Centre Koper, Garibaldijeva 1, SI-6000 Koper, Slovenia; gasper.kozlovic@zrs-kp.si (G.K.); milena.bucarmiklavcic@zrs-kp.si (M.B.-M.); 3 Faculty of Medicine, University of Rijeka, Braće Branchetta 20, HR-51000 Rijeka, Croatia; olivera.koprivnjak@medri.uniri.hr

**Keywords:** olive fruits, harvesting method, olive oil quality, FAEE, waxes, fatty acids, volatile compounds, phenolic compounds, sensory characteristics

## Abstract

Increasing competition in the olive oil market and labor shortages have accelerated the use of mechanical harvesting, raising concerns about potential fruit damage and its impact on oil quality. This study examined how three harvesting methods: manual using hand-held combs (B-HH) and two mechanical, hand-held shaker rake (B-MH-1) and self-propelled trunk shaker (B-MH-2), affect the quality and composition of Buža variety virgin olive oil. The greatest damage to the fruits occurred in B-MH-1, whereas the least was observed in B-HH. Olives were processed within 24 h, and oils were analyzed for basic quality parameters, fatty acid ethyl esters (FAEE), waxes content, fatty acid composition, volatile and phenolic profiles, and sensory attributes. Harvesting method did not significantly affect acidity, peroxide value, UV indices, FAEE, waxes, and fatty acids. Analyses of volatile and phenolic compounds revealed only slight differences. Nevertheless, sensory assessment detected no defects, with only minor reductions in positive odor attributes in B-MH-1. Taste attributes remained unchanged, consistent with similar total phenolic content. Overall, when olives are promptly processed, all investigated harvesting methods result in high-quality Buža olive oil.

## 1. Introduction

Virgin olive oil (VOO) has long been a defining element of Mediterranean agriculture and culture, valued for its nutritional properties and distinctive flavor and aroma. The highest quality category, extra virgin olive oil (EVOO) [1,2], reflects both the quality of the fruit and the care taken during oil production. To preserve freshness and prevent degradation of the fruits, it is recommended to process olives within 24 to 48 h after harvest. Longer delays can promote fermentation and loss of quality [3]. Therefore, the way olives are harvested and handled before milling can directly influence oil quality [4,5].

Harvesting is one of the most demanding and costly operations in olive production. Depending on orchard type, topography, and canopy structure, producers can choose manual, semi-mechanical, or fully mechanical methods [4,6]. Traditional manual harvesting, where fruits are picked by hand or with small combs, minimizes mechanical stress and helps preserve the integrity of the olive, resulting in high oil quality [4,7]. However, this method is extremely labor-intensive and time-consuming, accounting for up to 50–60% of total production costs [6,8]. In recent years, due to labor shortages and increasing costs, attention has shifted toward mechanized harvesting [9]. Systems such as hand-held electric or pneumatic combs, trunk and branch shakers, and over-the-row (straddle) harvesters designed for super-intensive orchards have made olive harvest faster and more efficient [4,10]. It is also important to ensure that these innovations preserve the highest possible VOO quality by minimizing fruit damage during harvesting. However, mechanical harvesting may cause bruising and softening of the pulp, rupture of cells, and increased fruit respiration, all of which can accelerate oil degradation [3,5].

The response of olives to mechanical harvesting depends greatly on the variety and the equipment used. Taiti et al. [11] reported that fruits of different varieties (Frantoio and Moraiolo) showed different sensitivity to bruising caused by harvesting with a hand-held shaker. Other authors have also reported that both the variety and the degree of fruit ripening influence the sensitivity of fruits to bruising caused by hand-held harvesting machines [12]. Bruising activates polyphenol oxidase, the enzyme responsible for the oxidation of polyphenols and the formation of brown pigments [13]. Research has shown that harvesting with hand-held shakers or electric combs causes more bruising than gentle manual picking [11,12]. In Arbequina olives, mechanical harvesting with over-the-row machines has been associated with higher peroxide value (PV) and free fatty acidity (FFA), as well as lower polyphenol content, compared to hand harvesting [3,14]. These results suggest that mechanical stress can trigger oxidative and hydrolytic processes that compromise oil quality, especially if the olives are not processed promptly after collection. Mechanical stress can also alter the lipoxygenase (LOX) pathway, which generates key volatile compounds, C5 and C6 aldehydes, alcohols, and esters, that give EVOO its fresh, green, and fruity aroma [15]. Improper handling or storage of damaged olives can prematurely activate this pathway, leading to altered volatile profiles and reduced aroma complexity [16].

Previous research on harvesting has mainly been focused on basic quality indices such as FFA, PV, and UV spectrophotometric parameters [14,17,18]. However, only a few recent studies have examined how different harvesting techniques affect phenolic and volatile composition [5,7], and even fewer have explored their influence on sensory characteristics mostly comparing manual and straddle harvesting [14,16]. This gap highlights the need for a more comprehensive understanding of how different harvesting techniques influence the quality, chemical composition and sensory properties of EVOO.

The concentration of fatty acid ethyl esters (FAEE) is a quality and authenticity criterion for the EVOO category, officially recognized in 2013 by the European Commission and implemented in international trade standards [1,2,19]. The formation of ethyl esters of fatty acids is closely related to fruit fermentation and degradation processes, typically occurring when damaged or overripe olives undergo microbial activity or ethanol formation prior to extraction [19]. Elevated FAEE levels indicate compromised raw material quality, even when other parameters remain within acceptable limits. Despite their importance, the effect of harvesting methods on FAEE content has not yet been investigated, representing a knowledge gap in understanding how mechanical and manual practices influence oil quality.

Another group of compounds relevant to the authenticity of VOO are the waxes [1,2], which originate from the olive fruit’s natural cuticular layer. It can be assumed that an additional portion of the wax fraction may end up in the oil as a consequence of fruit skin damage that occurs during the harvesting process. However, the potential relationship between harvesting techniques and wax content in olive oil remains unexplored.

In addition to these markers, the fatty acid composition of olive oil, particularly the relative proportions of monounsaturated, polyunsaturated, and saturated fatty acids, is a key determinant of nutritional value, oxidative stability, and authenticity [1,20]. To the best of our knowledge, this is the first study to investigate how different harvesting methods affect the fatty acid composition of virgin olive oil.

Olive production in Croatia is mainly concentrated in the Mediterranean region along the Adriatic coast. Olives are grown in both traditional and intensive groves covering a total area of 18,370 ha, with a total olive fruit yield of 40,112 t in 2022 [21]. Among the many autochthonous olive varieties, the Buža variety is one of the most common in the Istria region (Croatia), renowned for producing oils with exceptional sensory characteristics [22]. Buža fruits are large, weighing 4–6 g [23], and are particularly sensitive to mechanical damage because of the structural properties of their tissue [24].

Therefore, the aim of this research was to evaluate how different olive harvesting methods influence the quality and composition of Buža olive oil. Methods representing the most common practices in traditional and intensive olive orchards were studied: manual harvesting using hand-held combs and two mechanized methods—one with a hand-held shaker rake and another with a self-propelled trunk shaker. Gentle hand harvesting of healthy fruits generally produces high-quality oil. However, because it is rarely used in practice due to high labor costs, this study considers a more realistic harvesting scenario. The research included analysis of the basic chemical parameters of oil quality, FAEE, waxes, fatty acid profile, phenolic and volatile compound composition and sensory properties of oils obtained from fruit harvested by the three different systems. Considering the above-mentioned gaps, the novelty of this research lies in providing the first comprehensive assessment of how different harvesting methods influence the formation of fatty acid ethyl esters, waxes, and the fatty acid profile in virgin olive oil, while also offering new insights into their impact on the oil’s sensory properties and related chemical constituents, namely volatile and phenolic compounds. By addressing these previously unexplored aspects, this study contributes to a deeper understanding of the effects and potential for optimizing mechanized harvesting to achieve high oil quality standards while improving economic efficiency.

## 2. Materials and Methods

### 2.1. Olive Samples and Harvesting Procedures

The research was carried out in the southern part of the Istrian peninsula, near the city of Vodnjan (Istria, Croatia) on 13-year-old olive (*Olea europaea* L.) trees of the Buža variety, spaced 6.0 × 6.0 m and trained to vase. Olives were collected on 7 October 2023. Three harvesting systems were used: (1) manual harvesting with hand-held combs (B-HH); (2) mechanical harvesting with an electric hand-held shaker rake with telescopic handles 2.3 m in length (Olivion P230, Pellenc SAS, Pertuis, France) (B-MH-1); and (3) mechanical harvesting with a self-propelled trunk shaker (Buggy 5000S, Pellenc SAS, Pertuis, France) (B-MH-2) For B-MH-2, automatic program 4 was used, as it has been employed for harvesting the Buža variety for years. For all harvesting methods, the same plastic nets placed under the trees were used for olive collection.

Harvesting was conducted on three olive trees of similar age and cultivated under the same agroecological conditions, from which 3 kg of olives were harvested using each method. Each tree served as one replicate in the experiment. To minimize the accumulation of mechanical damage, the harvest began with hand-held combs, followed by branch shaker harvesting, and concluded with trunk shaker harvesting. During both hand harvesting with combs and mechanical harvesting with hand-held shakers, olives were collected from all sides and heights of the tree to ensure representative sampling. The harvested fruits from each tree for each harvesting method were mixed to obtain a homogeneous sample. In total, nine olive samples were collected (three harvesting methods, three trees).

The ripening stage of the fruits was approximately uniform across all trees at the time of harvest. The maturity index (MI) was calculated immediately after harvest according to the Guide for the determination of the characteristics of oil-olives [25] and it was 1.8 ± 0.1 across all treatments.

The fruits examined were in good condition, with no visible signs of damage from abiotic factors (e.g., hail) or biotic factors (such as diseases or pests). All observed damage to the harvested fruit was caused by the harvesting systems used in the research. Surface damage to the olives was assessed visually after 24 h when the damage was clearly noticeable. Based on the extent of the damage, 100 olives were categorized into two groups: (1) undamaged, consisting of olives with no visible damage, and (2) damaged, comprising olives with any visible external damage resulting from the harvesting process. The percentage of damaged fruits was calculated according to the procedure described by [14].

### 2.2. Post-Harvest Handling and Processing

Olives from each harvesting treatment and replicate were transported to the laboratory in separate ventilated plastic crates and maintained at a temperature of 20 ± 2 °C during storage. All olive samples (2 kg per sample) were processed separately into olive oil under identical conditions within 24 h after harvesting. Olive oil was produced using an Abencor laboratory-scale two-phase system (MC2 Ingeneria y Sistemas, Seville, Spain). Olives were crushed using a hammer mill, and the olive paste was malaxed under controlled conditions (35 min at 25 ± 1 °C). Olive oil was separated by centrifugation (1 min at 3500 rpm) and subsequently clarified through an additional centrifugation step (2 min at 4500 rpm) using Universal 320 R (Hettich, Tuttllingen, Germany) centrifuge. The oil obtained from each tree and each harvesting method was processed and stored separately in dark glass bottles at controlled room temperature (18 ± 2 °C) until analysis.

### 2.3. Analysis of Olive Oil

#### 2.3.1. Basic Quality Parameters of Virgin Olive Oils

Basic quality parameters of virgin olive oils, free fatty acids, peroxide value and spectrophotometric indices (K_232_, K_270_, and ΔK) were measured according to International Olive Council (IOC) regulations [26,27,28].

#### 2.3.2. Fatty Acid Ethyl Esters and Waxes

The determination of waxes and FAEEs in olive oil samples (expressed as mg/kg of oil) was conducted according to the standard IOC method [29]. Analyses were performed using a Varian 3350 gas chromatograph (Varian Inc., Harbour City, CA, USA) equipped with a flame ionization detector (FID) and a 15 m metal capillary column (MXT-5, Restek, Bellefonte, PA, USA) coated with Crossbond 5% diphenyl/95% dimethylpolysiloxane (0.25 mm internal diameter, 0.25 μm film thickness). Methyl heptadecanoate and lauryl arachidate (≥99% purity; Sigma, St. Louis, MO, USA) were used as internal standards.

#### 2.3.3. Analysis of Fatty Acid Methyl Esters (FAME)

FAME analysis was performed according to the standard IOC method [30] using a Varian 3350 GC (Varian Inc., Harbour City, CA, USA) equipped with an FID and an Rtx-2.330 capillary column (105 m length, 0.25 mm i.d., 0.25 m f.t.; Restek, Bellefonte, PA, USA). FAMEs in oils were identified based on their retention times relative to a standard FAME mixture (Sigma, Darmstadt, Germany) and according to the reference method [30]. Relative amounts were expressed as proportions (%) of total fatty acids.

#### 2.3.4. Volatile Compound Analysis

Volatile compounds were determined by headspace solid-phase microextraction coupled with gas chromatography-flame ionization detection and mass spectrometry (HS-SPME-GC-FID/MS) according to the IOC method [31]. The analytical procedure was performed using a gas chromatograph (Agilent 7890B, Agilent Technologies, Shanghai, China) equipped with a Gerstel MPS robotic autosampler, flame ionization detector and a mass selective detector (Agilent 5977C MS, Agilent Technologies, Shanghai, China). The system was configured with an auxiliary electronic pressure control module enabling splitting of the column effluent for simultaneous detection by both detectors. Quantification was performed using the FID signal, while compound identification was based on mass spectral from the MS detector. Separation was achieved on a Phenomenex ZB-WAX capillary column with a polyethylene glycol stationary phase (60 m length, 0.25 mm inner diameter, 0.25 μm film thickness; Phenomenex Inc, Torrance, CA, USA). Solid-phase microextraction was performed using a StableFlex fiber assembly coated with divinylbenzene/carboxen/polydimethylsiloxane (DVB/CAR/PDMS, Agilent 5191–5874, Agilent Technologies, Shanghai, China).

Oil samples (1.9 g) were placed in 20 mL headspace vials and spiked with 4-methyl-2-pentanol (0.1 g) as an internal standard. Samples were equilibrated at 40 °C for 10 min with agitation at 250 rpm, followed by 40 min SPME fiber exposure in the headspace. Thermal desorption was performed in the GC injection port at 250 °C for 5 min in splitless mode. Hydrogen was used as the carrier gas at a flow rate of 1.5 mL/min. The oven temperature was held at 40 °C for 10 min, then increased at 3 °C/min to 200 °C, and finally ramped to 250 °C and held for 5 min for column cleaning. The FID operated at 260 °C, with hydrogen flow at 30 mL/min, air at 300 mL/min, and nitrogen as makeup gas at 25 mL/min. Quantification was performed using internal standard calibration with 4-methyl-2-pentanol (Sigma-Aldrich, Buchs, Switzerland, ≥97.5%) and external calibration curves prepared in refined olive oil matrix. Calibration standards were prepared at two concentration ranges: 0.05–10.00 mg/kg for low concentration analytes and 0.20–25.00 mg/kg for high concentration analytes. As described in the official IOC protocol [31], the analytical standards used were: octane (Supelco, Buchs, Switzerland, ≥99.7%), ethanol (Honeywell, Seelze, Germany, ≥99.8%), 3-methyl-1-butanol (Supelco, Buchs, Switzerland, ≥98.5%), propanoic acid (Supelco, Buchs, Switzerland, ≥99.5%), 6-methyl-5-hepten-2-one (Sigma-Aldrich, Milwaukee, WI, USA, ≥98.5%), acetic acid (Honeywell, Seelze, Germany, ≥99.8%), ethyl acetate (Honeywell, Seelze, Germany, ≥99.5%), (*E*)-2-heptenal (Sigma-Aldrich, Milwaukee, WI, USA, ≥95.0%), 1-octen-3-ol (Sigma-Aldrich, Steinheim, Germany, ≥98.0%), ethyl propanoate (Sigma-Aldrich, Steinheim, Germany, ≥98.5%), hexanal (Supelco, Buchs, Switzerland, ≥95.0%), nonanal (Supelco, Laramie, WY, USA, ≥99.5%), (*E*,*E*)-2,4-hexadienal (Sigma-Aldrich, Stenheim, Germany, ≥94.0%), (*E*)-2-decenal (Supelco, Buchs, Switzerland, ≥96%), pentanoic acid (Supelco, Buchs, Switzerland, ≥99.8%), (*E*)-2-hexenal (Sigma-Aldrich, Stenheim, Germany, ≥98.0%), (*Z*)-3-hexenyl acetate (Supelco, Buchs, Switzerland, ≥98.0%), and 1-hexanol (Supelco, Buchs, Switzerland, ≥99.9%). All samples were analyzed in triplicate.

#### 2.3.5. Phenolic Compound Analysis

The composition of phenolic compounds in olive oil samples from this experiment was determined using the standard IOC method [32]. This method involves extracting the polar phenolic fraction from 2 g of oil by adding 1 mL of internal standard solution (syringic acid, 0.015 mg/mL) and a mixture of methanol and water (5 mL; 80:20, *v*/*v*). The procedure included mixing for one minute, sonication for 15 min, and centrifugation at 4500 rpm for 25 min, followed by analysis of the extract by liquid chromatography with a diode array detector (HPLC-DAD). Chromatographic separation was performed using an Agilent Infinity 1260 liquid chromatograph equipped with a G1311B quaternary pump, G1329B autosampler, G1316A column oven, and G4212B DAD detector (Agilent Technologies, Palo Alto, CA, USA), and an octadecyl silica (C18) reverse-phase column, Agilent Omnispher CP27835 (4.6 mm × 250 mm, 5 μm, pore 110 Å, Agilent Technologies, Santa Clara, CA, USA). The mobile phase consisted of (A) 0.2% ortho-phosphoric acid in HPLC grade water and (B) methanol/acetonitrile (1:1, *v*/*v*). Ortho-phosphoric acid (85%) and HPLC grade solvents acetonitrile, methanol, and water were obtained from Sigma-Aldrich (St. Louis, MO, USA). The solvent gradient started with 4% B, gradually increasing to 50% B over the first 40 min; from 40 to 45 min to 60% B; from 45 to 60 min to 100% B; held isocratic at 100% B from 60 to 70 min; then returned to the starting composition of 4% B over 70 to 72 min; and finished with 10 min under isocratic conditions at 4% B. The injection volume was 20 μL. The flow rate was 1.0 mL/min at 25 °C. UV/VIS detection was performed at 280 nm for all phenols except for flavonoids, which were quantified at 350 nm. Stock solutions of the phenolic standards were prepared in methanol (80%, *v*/*v*), except for oleacein and oleocanthal, which were prepared in acetonitrile.

Identification was performed by comparing retention times and typical maximum absorbance wavelengths from the standardized method, as well as by comparing UV/V is spectra recorded from 200 to 600 nm with those of pure chemical standards when available, or with other literature sources [33]. Phenolic compounds were quantified using the internal standard method with syringic acid (Sigma-Aldrich Chemie GmbH, Steinheim, Germany). Calculated concentrations were expressed in mg/kg of oil. For identification purposes, standard solutions of pure phenols in methanol were prepared: hydroxytyrosol and oleuropein from Extrasynthesis (Genay, France), tyrosol, apigenin, vanillic and *p*-coumaric acid from Fluka (Buchs, Switzerland), vanillin and pinoresinol from Sigma-Aldrich-Merck KGaA (Darmstadt, Germany), and luteolin from Cayman Chemical Co. (Ann Arbor, MI, USA).

Quantification was performed semi-quantitatively using the response factor ratio of tyrosol (Fluka, Buchs, Switzerland) relative to syringic acid at 280 nm. Based on this, the final results for total and individual phenols were expressed in mg/kg as tyrosol, with syringic acid used as the internal standard. The main phenolic compounds belonged to the secoiridoid group: oleuropein; oleuropein aglycone (dialdehyde, aldehyde, and hydroxyl forms); ligstroside aglycone (dialdehyde, aldehyde, and hydroxyl forms); decarboxymethyl oleuropein aglycone, dialdehyde form (3,4-DHPEA-EDA), also known as oleacein; and decarboxymethyl ligstroside aglycone, dialdehyde form (*p*-HPEA-EDA), also known as oleocanthal. Other phenolic compounds were from the groups of simple phenols (tyrosol, hydroxytyrosol, hydroxytyrosyl acetate, tyrosyl acetate, vanillin), phenolic acids (vanillic acid, caffeic acid, ortho- and para-coumaric acid, cinnamic acid), flavonoids (luteolin, apigenin, methyl-luteolin), and lignans (pinoresinol and 1-acetoxy-pinoresinol, which were not chromatographically separated).

#### 2.3.6. Sensory Evaluation

Sensory evaluation of the VOO samples was carried out by the sensory panel of the Institute of Agriculture and Tourism (Poreč, Croatia), which has been continuously recognized by the IOC since 2014 for the sensory assessment of VOOs. The panel consisted of eight trained assessors (four male and four female, average age 42 years). Informed consent was obtained from all assessors prior to participation. A quantitative descriptive sensory analysis was performed following the IOC protocol [34], using an evaluation sheet with a 10 cm unstructured intensity scale (0 cm indicating absence of the attribute; 10 cm indicating maximum perceived intensity) for both aroma and taste descriptors. The evaluation form was further extended to include additional taste attributes (sweet, astringent) and additional aroma descriptors (green grass/leaf, apple, tomato leaf, almond, aromatic herbs, radicchio, peas). These attributes were added to the scale to obtain a more detailed sensory characterization of the samples.

#### 2.3.7. Statistical Analysis

The results are presented as mean values of three technical repetitions ± standard deviations. A one-way analysis of variance (ANOVA) was applied to evaluate differences among samples at a 5% significance level. Homogeneity of variances was verified using Levene’s test, and mean comparisons were performed with Tukey’s honest significant difference (HSD) test at *p* ≤ 0.05. The strength and direction of linear relationships were assessed using Pearson’s correlation coefficient (r). Pearson’s correlation analysis was used to investigate linear relationships between the percentage of damaged fruits and the concentrations of volatile and phenolic compounds, with statistical significance set at *p* < 0.05. Additionally, grouping of samples obtained by different harvesting methods was performed using an unsupervised multivariate statistical analysis method, Principal Component Analysis (PCA). The variables selected were compounds that, according to analysis of variance, showed statistically significant differences with respect to the harvesting method. All statistical analyses were conducted using the Statistica software package, version 13.2 (StatSoft Inc., Tulsa, OK, USA).

## 3. Results and Discussion

### 3.1. Fruit Damage

**Figure 1:**
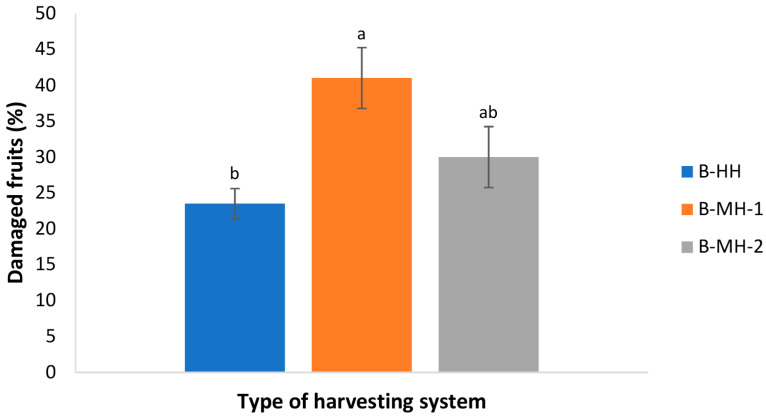
Percentage of the damaged Buža variety fruits related to the harvesting system (B-HH: manual harvesting with hand-held combs; B-MH-1: harvesting with an electric hand-held shaker rake; B-MH-2: mechanical harvesting with a self-propelled vibrator). Results represent mean values of three technical repetitions ± standard deviations. Different letters above bars represent significant differences among the treatments (Tukey’s test, *p* < 0.05) for each parameter separately.

The harvesting system affected both the extent and type of fruit damage. The highest damage level (41.0 ± 4.24%) was observed in fruits harvested with hand-held shaker rakes, while the lowest (23.5 ± 2.12%) occurred in fruits collected with a hand-held comb (Figure 1). Famiani et al. [7] also reported that hand-held combs caused less damage than hand-held machines and trunk shakers, while Jiménez-Jiménez et al. [35] found that trunk shakers caused bruising up to twelve times higher than the manual method.

The susceptibility of olive fruit to mechanical damage changes during ripening. Green, immature olive fruits are harder and less susceptible to mechanical damage [36], which is visually more detectable than on ripe fruits. To avoid the influence of ripeness, we used fruit of uniform maturity at the typical harvest stage for the Buža variety (MI = 1.8 ± 0.1).

**Figure 2:**
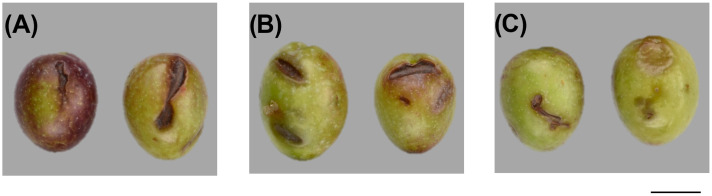
Characteristics of Buža variety fruit damage: (**A**) manual harvesting with hand-held combs; (**B**) harvesting with an electric hand-held shaker rake; (**C**) mechanical harvesting with a self-propelled vibrator (bar represents 1 cm).

Regarding the type of fruit damage, hand-held combs caused grooved damage from branch notches during pulling (Figure 2A) while hand-held shakers produced elongated depressions from impacts with plastic comb parts (Figure 2B). The mechanical trunk shaker caused damage from leaf and branch notches and from the vigorous impact of the fruit on the net on the ground (Figure 2C).

### 3.2. Oil Quality Parameters

No significant differences were observed in the basic quality parameters of olive oils obtained from Buža fruits harvested using three methods: manual harvesting with a hand-held comb (B-HH), mechanical harvesting with a hand-held shaker rake (B-MH-1), and mechanical harvesting with a self-propelled trunk shaker (B-MH-2) (Table 1).

The FFAs, PV, and UV spectrophotometric absorbance indices were all within the limits established for EVOO [1], regardless of the harvesting technique. FFAs are formed when lipolytic enzymes in olive pulp and seeds hydrolyze the oil after the fruit’s cells are damaged. High FFA levels result mainly from unhealthy, bruised, poorly stored, and long-stored olives [37]. The low level of FFAs determined in oils from all three harvesting methods, despite differences in the extent and type of fruit damage (Table 1), indicates the importance of urgent fruit processing (in this study, within 24 h after harvest). This agrees with Famiani et al. [7], who observed no effect of harvesting systems on FFA when fruits of Arbequina cv. and Frantoio cv. were processed immediately, while FFA increased after one week of fruit storage. This was particularly evident in oils from more severely damaged Arbequina fruits harvested with hand-held machines or straddle machines compared to fruits collected by hand-held combs.

Similarly, oxidation parameters (PV and UV indices) showed no differences among harvesting methods in several studies when fruits of the cultivars Frantoio [5,7], Arbequina [7], or Leccino [38] were processed promptly. However, some authors reported slight increases in FFA and PV after mechanical harvesting of the cultivars Gemlik and Ayvalik [3] or Arbequina [18]. These inconsistencies likely reflect interactions between the harvesting system and cultivar-specific characteristics. Studies comparing different varieties [7,39] confirm that varietal traits strongly influence how the harvesting method affects olive oil quality.

### 3.3. Fatty Acid Ethyl Esters

**Figure 3:**
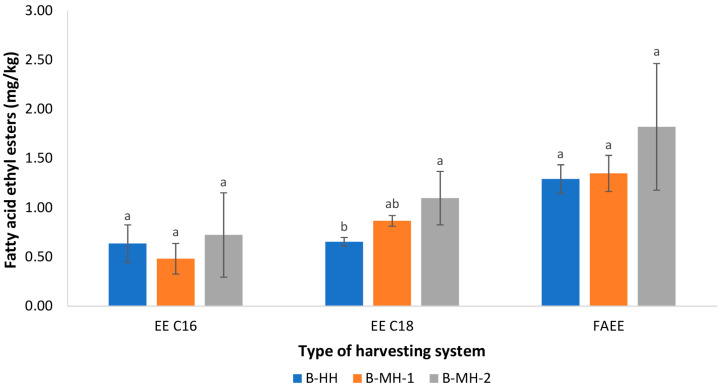
Concentration (mg/kg) of fatty acid ethyl esters (FAEE) of Buža virgin olive oils obtained from fruits harvested using three different methods: manual harvesting with hand-held combs (B-HH), mechanical harvesting with hand-held shaker rake (B-MH-1), and mechanical harvesting with a self-propelled trunk shaker (B-MH-2). Results represent mean values of three technical repetitions ± standard deviations. Different letters above bars represent significant differences among the treatments (Tukey’s test, *p* < 0.05) for each parameter separately. FAEE represents the sum of ethyl palmitate (EE C16) and ethyl stearate (EE C18).

High concentrations of FAEEs in virgin olive oil are generally associated with low-quality fruits that have undergone hydrolytic and fermentative processes before extraction, resulting in increased formation of FFAs and alcohols (primarily ethanol and methanol) [19]. Due to their strong link to fermentative deterioration, FAEEs have been recognized as key olive oil quality marker [1]. In our study, the only statistically significant difference was observed for ethyl stearate levels (EE C18), which were the lowest for hand-held combs (B-HH) and the highest for trunk-shaker samples (B-MH-2) (Figure 3). However, because the levels of FAEE precursor, FFA (Table 1) did not differ among treatments, it is reasonable to assume that the observed differences in EE C18 between B-HH and B-MH-2 are not a direct consequence of the harvesting method. The absence of differences in FAEE among harvesting methods could therefore be attributed to the short interval between harvest and processing, which was likely insufficient to allow ethanol accumulation and subsequent FAEE formation, even in mechanically damaged fruits. Moreover, FAEE concentrations in all tested samples did not exceed the maximum limit established for the EVOO category according to international trade standards [1].

### 3.4. Waxes

**Figure 4:**
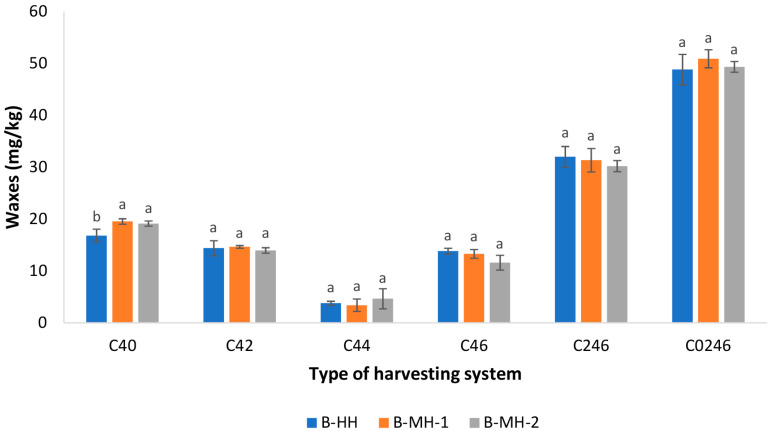
Concentrations (mg/kg) of individual wax esters (C40, C42, C44, and C46) as well as C246 (sum of C42, C44 and C46 waxes) and C0246 (sum of C40, C42, C44, and C46) of Buža virgin olive oils obtained from fruits harvested using three different methods: manual harvesting with hand-held combs (B-HH), mechanical harvesting with hand-held shaker rake (B-MH-1), and mechanical harvesting with a self-propelled trunk shaker (B-MH-2). Results represent mean values of three technical repetitions ± standard deviations. Different letters above bars represent significant differences among the treatments (Tukey’s test, *p* < 0.05) for each parameter separately.

The olive fruit skin consists of a layer of epidermal cells covered by a cuticle with a fatty–waxy coating that contains approximately 10% waxes. A portion of these compounds is transferred into the oil during extraction, as the fruit surface comes into direct contact with the oil phase. During extraction of residual oil from olive pomace with organic solvents, a significant amount of wax esters is extracted with the oil. For this reason, the wax content serves as an authenticity criterion indicating the presence of olive pomace oil in virgin olive oil. When assessing compliance with the EVOO and VOO quality categories, the sum of the C42, C44, and C46 waxes (C246) is considered, with a maximum limit of 150 mg/kg [1,40]. It can be assumed that the transfer of wax esters to the oil may be facilitated by damage to the fruit skin during the harvesting. To our knowledge, this study is the first to examine how different olive harvesting methods affect wax content in virgin olive oil. While evaluating the differences in these compounds among treatments, a statistically significant difference was observed only for C40, a wax ester not considered an authenticity criterion (Figure 4), with the lowest value found in the hand-harvested samples (B-HH). Other individual waxes as well as the sum C246 showed no statistically significant differences. These results indicate that, under the applied harvesting and processing conditions, mechanical damage did not significantly enhance wax migration into the oil. Therefore, in all investigated samples, the sum of C246 waxes was below the maximum limits established for the EVOO and VOO categories, while the total amount of waxes (C0246) was far below the limit defined for the lampante olive oil category [1].

### 3.5. Fatty Acids

The fatty acid composition of Buža virgin olive oils was not markedly affected by the harvesting method, with only minor variations observed among treatments (Table 2). In all samples, the fatty acid profiles were within the limits established for the EVOO category [1,2]. Oleic acid was the most abundant fatty acid, followed by palmitic acid and linoleic acid (Table 2). This composition corresponds to the typical VOO rich in oleic acid, which is a key determinant of its nutritional quality and oxidative stability [20]. No significant differences in oleic acid content were observed among harvesting methods. Among saturated fatty acids (SFA), palmitic acid was predominant, while stearic acid and arachidic acid were present in moderate amounts (Table 2). A significant difference was observed only for arachidic acid, which was slightly lower in oils obtained by B-MH-2 compared to B-HH and B-MH-1. However, these variations were quantitatively negligible and did not compromise compliance with regulatory standards for the EVOO category [1]. The MUFA/PUFA ratio in Buža oils was approximately 5.3–5.4, which is favorable for both oxidative stability and health-promoting properties of the oils [41]. These findings confirm the high nutritional quality of Buža oils, regardless of harvesting method. Overall, the results indicate that the method of fruit harvesting has negligible influence on the fatty acid composition of Buža oils. The fatty acid profile appears to be a stable, variety dependent characteristic that is not meaningfully influenced by technological factors such as the harvesting method.

### 3.6. Volatile Compounds

Significant differences were observed in the concentrations of certain volatile compounds in Buža virgin olive oils obtained using the three harvesting methods (Table 3). Ethyl acetate and 3-methyl-1-butanol, both typically associated with fermentation notes [15,42], had slightly higher concentrations in oils produced from fruits harvested with the trunk shaker (B-MH-2) than in the other two treatments. A similar trend was observed for (*E*)-2-heptenal and 6-methyl-5-hepten-2-one, both associated with fermentative defects in olive oils [43,44]. Despite their presence, the concentrations of ethyl acetate, 3-methyl-1-butanol and 6-methyl-5-hepten-2-one were substantially below their odor threshold values [44], while the (*E*)-2-heptenal concentration was near its odor threshold value (0.042 mg/kg [44]). These results suggest that none of the harvesting methods examined substantially contribute to the formation of fermentative volatiles.

Pentanoic acid, associated with rancid and fermentative notes [43,44], was influenced by harvesting, with the lowest concentration found in B-HH, the highest in B-MH-1, and an intermediate level in B-MH-2. This result was consistent with the greater degree of fruit damage observed in B-MH-1 (Figure 2). Pearson’s correlation analysis showed a strong positive correlation between the percentage of fruit damage and pentanoic acid concentration (r = 0.896, *p* < 0.05). However, the concentration of pentanoic acid was below its odor threshold [44] and most likely had no impact on the sensory assessment (Figure 3).

The concentration of hexanal, associated with apple and green grass notes [15,44] was highest in B-HH oils and significantly lower when mechanization was used, especially in B-MH-2. In contrast, the concentration of 1-hexanol, which contributes to fruity and banana notes [15,43], was significantly higher in oils obtained from mechanical harvested fruits (B-MH-1 and B-MH-2), compared to those from olives harvested manually (B-HH). Pearson’s correlation analysis showed a strong positive correlation between the percentage of fruit damage and 1-hexanol concentration (r = 0.841, *p* < 0.05). These results suggest enhanced lipoxygenase pathway activity under mechanical stress [45] and promoted conversion of hexanal into the corresponding alcohol through the action of alcohol dehydrogenase (ADH), resulting in increased levels of 1-hexanol. Similar findings related to 1-hexanol and harvesting method were also reported by other authors [5,7]. Corti et al. [5] found that 1-hexanol increased in olive oil samples obtained from Frantoio cv. fruits harvested by hand-held electric combs with vibrating systems compared to those obtained after manual harvest. C6 alcohol levels were also higher in Frantoio cv. oils obtained by hand-held machines and trunk shakers compared to gentle hand harvest and hand-held combs [7]. (*E*)-2-Hexenal, the dominant volatile in VOOs and a contributor to green grass and green apple aromas [15,42,43], had the lowest concentration in B-MH-1, where the highest percentage of damaged fruits was found. For the Arbequina and Frantoio cultivars, Famiani et al. [7] also reported that increased fruit damage is associated with lower levels of C5 and C6 aldehydes and (*E*)-2-hexenal. According to Morales-Sillero et al. [16], hand harvesting results in the highest C6 volatile concentrations, while mechanical harvesting with a grape straddle harvester reduced values by 40%. The reduction in C6 aldehyde concentration may be associated with increased ethylene biosynthesis in damaged fruits, which leads to accelerated ripening. Elevated ethylene production, induced by mechanical injury, can alter the activity of enzymes involved in the LOX pathway and related volatile compound metabolism, leading to a shift in the balance of aldehydes and other aroma-related molecules [7,16]. However, in the present study, Pearson’s correlation analysis did not reveal a statistically significant negative linear association between the percentage of fruit damage and the concentration of (*E*)-2-hexenal (*p* < 0.05).

### 3.7. Phenolic Compounds

The total phenolic content (TPC) of Buža virgin olive oils was not significantly affected by the harvesting method (Table 4). Although oils obtained by mechanical harvesting with hand-held shakers showed slightly higher TPC compared to those from trunk shaker harvesting and manual harvesting with hand-held combs, these differences were not statistically significant.

The phenolic profile of the Buža virgin olive oil samples was consistent across all samples in this study, with only minor deviations among harvesting methods. In some cases, statistically significant differences were observed among certain phenolic compounds, despite the fact that their concentrations were generally low and varied within a narrow range. Similar trends have been reported in a previous study [7], where mechanical harvesting did not significantly alter TPC in Frantoio cv. oil compared to harvesting by hand-held combs, but differences in individual phenolic compounds were observed. Several studies have reported higher TPC in olive oils from hand-harvested fruits compared to those harvested mechanically by hand-held shaker [5,11,17]. This difference was attributed to fruit damage caused by mechanical harvesting, which accelerates enzymatic oxidation and phenolic degradation, leading to lower TPC in the resulting oils [5,11]. In our study, we did not investigate gentle manual harvesting, but rather assisted hand-harvesting by combs, which can also cause some degree of fruit damage similar to mechanical harvesting, although to a lesser extent (Figure 1 and Figure 2). The non-significant differences in TPC observed in the present study (Table 4) suggest that rapid processing limited oxidation, minimizing the impact of the harvesting method on TPC.

The main phenolic compounds responsible for the antioxidant activity and shelf life of VOO are secoiridoids, predominantly present as aglycones and other derivatives of oleuropein and ligstroside [46]. The final content and profile of secoiridoids reflect a balance between the hydrolysis of glucosides catalyzed by β-glucosidase, and oxidation, driven in tissue primarily by polyphenol oxidase and, to a lesser extent, by peroxidase activity [13,47]. Among the secoiridoids, the highest concentrations were observed in B-MH-1 oils. Notably, oleacein and oleuropein were more abundant in B-MH-1 compared to the other treatments, although these differences were not statistically significant. Since secoiridoids are mainly derived from the enzymatic hydrolysis of oleuropein and ligstroside during fruit processing, it is possible that, starting immediately from harvesting, the greater disruption of cellular tissue in B-MH-1 facilitated the interaction of phenols released from intracellular vacuoles with endogenous enzymes from the cytosol, resulting in aglycones and further oxidized forms [9,13]. The concentrations of the oxidized forms of oleacein and oleocanthal were elevated in B-MH-1 compared to the other two treatments, corresponding to the highest level of fruit damage determined in B-MH-1 (Figure 2). Pearson’s correlation analysis showed a moderate to strong positive correlation (*p* < 0.05) between the percentage of fruit damage and several secoiridoids, including the oxidized form of oleacein (r = 0.922), oleocanthal (r = 0.763), oxidized form of oleocanthal (r = 0.707), and oleuropein (r = 0.694). This is consistent with the well-established observation that cellular tissue disruption leads to the release of phenolic compounds and oxidative enzymes [13]. In contrast, B-MH-1 showed the lowest concentrations of the aldehydic and hydroxylic forms of the ligstroside aglycon. Pearson’s correlation analysis showed moderate negative correlation between the percentage of fruit damage and the aldehydic and hydroxylic forms of the ligstroside aglycon (r = 0.922, *p* > 0.05). Famiani et al. [7] reported a negative correlation between the levels of secoiridoid derivatives and the extent of fruit damage in Frantoio cv. The discrepancy between our results and those reported by Famiani et al. [7] in the trend of several secoiridoids may be attributed to differences in harvesting methods, the intrinsic properties of the olive variety, and the duration of fruit storage prior to processing into oil.

Among the simple phenols, significant differences were found between B-MH-1 and B-MH-2 for hydroxytyrosol and tyrosol acetate. These compounds were present at the highest concentrations in B-MH-1 and at the lowest concentrations in B-MH-2 samples (Table 4). Hydroxytyrosol in oil is primarily formed through the enzymatic or chemical degradation of oleuropein during oil extraction and early post-harvest handling [48], so its higher levels in B-MH-1 oils may reflect increased enzymatic transformation of oleuropein associated with greater fruit damage caused by hand-held shakers compared to other harvesting systems (Figure 2). Tyrosol acetate is an acetylated derivative of tyrosol, and the higher levels of tyrosol acetate in B-MH-1 oils suggest that mechanical stress from hand-held shakers may favor acetylation of tyrosol precursors. This assumption was supported by Pearson’s correlation analysis which showed strong positive correlation between the percentage of fruit damage and the concentration of tyrosol acetate (r = 0.779; *p* < 0.05).

Among the phenolic acids analyzed, vanillic + caffeic and *o*-coumaric acids showed the lowest concentrations in B-MH-2, with no significant differences between B-HH and B-MH-1. Cinnamic acid reached its highest concentration in B-MH-2. Pearson’s correlation analysis revealed no significant relationship (*p* < 0.05) between the percentage of fruit damage and the concentrations of these phenolic acids. Therefore, the observed variations in phenolic acid profiles among the oils are likely not attributable to mechanical stress during harvesting.

Lignans (pinoresinol and acetoxy-pinoresinol) showed comparable concentrations among the different harvesting methods, with no statistically significant differences. This observation is consistent with previous studies suggesting that lignans are less sensitive to technological or mechanical variables during oil extraction, probably due to their low antioxidant activity which makes them less susceptible to oxidation [49]. Similar results regarding lignans were reported by Famiani et al. [7] in Frantoio oils obtained using different harvesting systems.

Oils from B-MH-2 had the highest total flavonoid content, with luteolin and apigenin particularly abundant. Since flavonoids contribute to the antioxidant activity of virgin olive oil [50,51], this enrichment may represent a qualitative advantage of B-MH-2 oils. Pearson correlation analysis did not show a significant correlation between the percentage of fruit damage and flavonoids (*p* < 0.05).

Phenolic acids, lignans and flavonoids are products of the phenylpropanoid pathway, which is in counterbalance with the biosynthesis of secoiridoid-type phenolic molecules. Since both pathways start from tyrosine as the precursor molecule [52,53], it can be assumed that the differences found between harvesting methods, particularly for phenolic acids and flavonoids, could result from a possible redirection of phenol biosynthesis pathways toward the phenylpropanoid pathway instead of the biosynthesis of secoiridoids. However, the conclusions cannot be definitive, since the final concentrations of phenols in olive oil are the combined result of complex reactions, including biosynthesis, biotransformation, isomerization, and hydrolytic and oxidative degradation of phenolic compounds [48].

### 3.8. Principal Component Analysis (PCA)

In order to gain a better understanding of the relationships between different olive harvesting methods and changes in the concentrations of specific chemical compounds in the obtained oils, Principal Component Analysis (PCA) was carried out.

**Figure 5:**
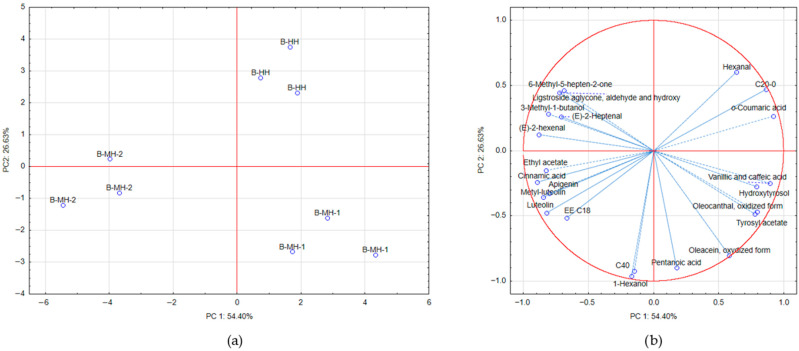
(**a**) Separation based on harvesting method in two-dimensional space defined by principal components PC1 and PC2 (unsupervised, PCA); (**b**) Projection of factor loadings of selected compounds along PC 1 and PC 2.

Successful grouping of samples from individual treatments along the first two principal components, PC1 and PC2, was achieved (Figure 5). The first two principal components together accounted for 81.03% of the total variance. The first principal component (PC1) explained 54.40% of the total variance, while additional separation of the samples was achieved along PC2, which accounted for 26.63% of the variance.

Among volatile compounds, olive oil samples B-MH-2, located in the Cartesian coordinate system with negative factor loadings along PC1, were characterized by lower concentrations of hexanal and higher concentrations of ethyl acetate, 3-methyl-1-butanol, (*E*)-2-hexenal, (*E*)-2-heptenal, and 6-methyl-5-hepten-2-one. Compared to B-HH and B-MH-1, B-MH-2 samples were characterized by lower levels of arachidic acid (C20:0) and the phenolic compounds hydroxytyrosol, tyrosol acetate, vanillic acid, caffeic acid, and *o*-coumaric acid, as well as by higher levels of all three flavonoid compounds and ligstroside aglycone, aldehydic and hydroxylated form.

Samples B-HH were clearly separated from samples obtained by mechanical harvesting, primarily along PC2, mainly due to lower values of wax esters C40 and the aroma compounds 1-hexanol and pentenoic acid. Samples B-MH-1 were distinguished in the fourth quadrant of the Cartesian coordinate system based on higher concentrations of phenolic compounds: vanillic + caffeic acid, simple phenols hydroxytyrosol and tyrosyl acetate, and oxidized phenolic forms of both oleacein and oleocanthal.

### 3.9. Sensory Characteristics

**Figure 6:**
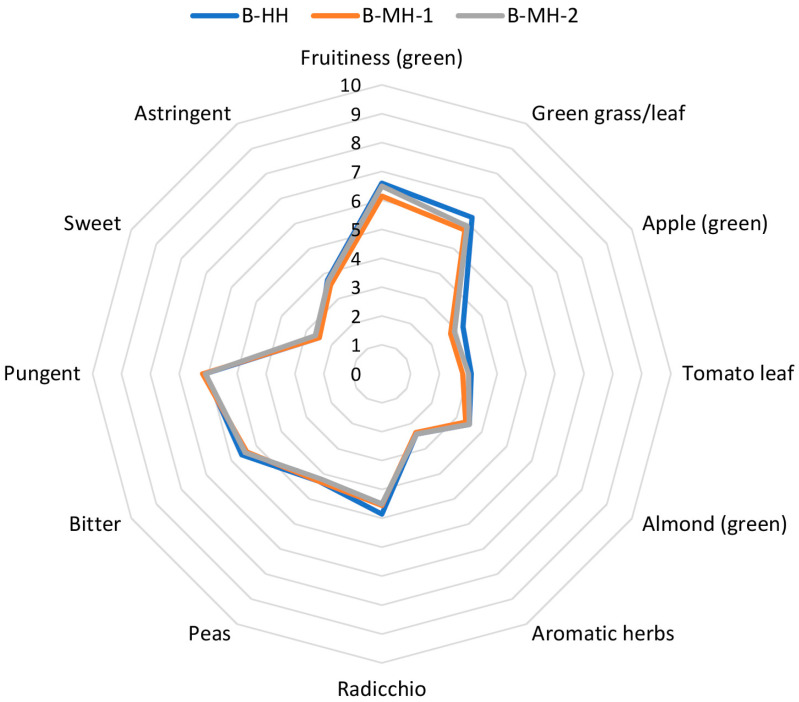
Results of descriptive sensory analysis of Buža virgin olive oils obtained from fruits harvested using three different methods: manual harvesting with hand-held combs, mechanical harvesting with hand-held shaker rake, and mechanical harvesting with a self-propelled trunk shaker. Results represent the mean values of medians of three technical repetitions for each sensory attribute.

Mechanical harvesting of Buža olives using hand-held shaker rake resulted only in a slight reduction in the intensity of green odor attributes in the produced VOO, particularly those associated with green fruitiness, green grass/leaf, green apple, and tomato leaf notes, compared with hand harvesting and harvesting using a self-propelled trunk shaker (Figure 6). The observed reduction in green odor attributes in Buža oils obtained by hand-held shakers (Figure 6), can likely be attributed to fruit damage, which was visually most pronounced in this harvesting method compared to two other harvesting methods (by hand-held combs and with trunk shakers) (Figure 1). The slight reduction in green sensory descriptors in B-MH-1 oils is consistent with the observed decrease in C6 aldehydes in the same samples (Table 3). Morales-Sillero et al. [16] also reported a decrease in intensity of positive sensory descriptors when grape straddle harvesters were used compared to gentle hand harvesting indicating that damage to olives during harvesting negatively affects the sensory profile of the resulting olive oils.

No significant differences were observed among the samples with respect to taste descriptors, bitterness or pungency (Figure 6), which is consistent with the similar total phenolic contents determined in these oils (Table 4). On the other hand, mechanical harvesting of Arbequina olives using an over-the-row harvester decreased bitterness, pungency and astringency, while increasing sweetness of the oil compared with hand harvesting [3,16]. These discrepancies in the results, besides the influence of variety and harvesting year, may partially be attributed to different levels of fruit damage caused by different harvesting systems used. Over-the-row harvesting has been reported to cause more damage to the fruit [7] than the harvesting method applied in this study.

## 4. Conclusions

This study provides a comprehensive assessment of how different harvesting methods, manual with hand-held combs and two mechanical methods, hand-held shaker rake and self-propelled trunk shaker, influence the quality and chemical composition of Buža virgin olive oil. The greatest fruit damage occurred when using the hand-held shaker rake, while the least damage was observed in fruits harvested with the hand-held combs.

A comparison of the different harvesting methods showed that when olives are processed within 24 h, the basic quality parameters of the oil (FFA, PV, and UV absorption indices), as well as FAEE, wax content, and fatty acid profiles, remain almost unaffected by the harvesting method. To the best of our knowledge, this is the first study to demonstrate that, under immediate processing conditions, different olive harvesting methods do not negatively influence FAEE concentrations, wax content, or the fatty acid composition of VOO. These findings provide new relevant insights into how mechanical and manual harvesting methods may affect chemical parameters related to oil quality and authenticity.

Analyses of volatile and phenolic compounds revealed only slight differences. None of the harvesting methods contributed substantially to the formation of fermentative volatiles. There was a strong positive correlation between 1-hexanol (fruity/banana notes) in relation to the degree of fruit damage, but the concentrations were below odor thresholds. Concentrations of (*E*)-2-hexenal (green grass or green apple) were above the odor threshold, but no linear association with the degree of fruit damage was found. Differences in total phenolic content among harvesting methods were not significant. Moderate to strong positive correlations were observed between the percentage of damaged fruits and several secoiridoids (oleacein, oleocanthal, their oxidized forms, and oleuropein) as well as tyrosol acetate. The aldehydic and hydroxylic forms of the ligstroside aglycon were negatively correlated with fruit damage. No significant correlations were found for phenolic acids, lignans, or flavonoids. Despite these variations, sensory evaluation did not detect any defects in the oils, and only a slight reduction in intensity of positive odor sensory attributes was observed in oils produced by mechanical harvesting. Taste attributes remained unchanged, consistent with similar total phenolic compounds determined in all samples.

Obtained results indicate that all investigated harvesting methods, when followed by prompt processing, are suitable for producing high quality VOO from the Buža variety. However, the observed influence of mechanical damage on volatile and phenolic profiles together with the potential for increased microbial activity in damaged fruits during extended storage prior to processing, raises concerns that olive oil quality could be negatively affected, highlighting the need for further investigation. Moreover, future studies should include additional olive varieties, as differences in fruit characteristics may result in varying responses to fruit damage during harvest. This study contributes to a deeper understanding of how harvesting can be optimized to preserve the high quality standards required for extra virgin olive oil production, while simultaneously ensuring the economic efficiency of the extra virgin olive oil production process.

## Figures and Tables

**Table 1:** Free fatty acids (FFA), peroxide value (PV), and ultraviolet spectrophotometric absorbance (K232, K268, ΔK) of Buža virgin olive oils obtained from fruits harvested using three different methods: manual harvesting with hand-held combs (B-HH), mechanical harvesting with hand-held shaker rake (B-MH-1), and mechanical harvesting with a self-propelled trunk shaker (B-MH-2).

Quality Parameters	B-HH	B-MH-1	B-MH-2	EVOO *
FFA (% oleic acid)	0.09 ± 0.01	0.09 ± 0.01	0.10 ± 0.01	≤0.80
PV (meq O_2_/kg)	5.67 ± 1.07	4.47 ± 0.35	5.43 ± 0.50	≤20.0
K_232_	1.99 ± 0.12	1.85 ± 0.10	2.14 ± 0.22	≤2.50
K_268_	0.13 ± 0.02	0.12 ± 0.01	0.15 ± 0.01	≤0.22
∆K	0.00 ± 0.00	0.00 ± 0.00	0.00 ± 0.00	≤0.01

Results represent mean values of three technical repetitions ± standard deviations. Different letters in a row represent significant differences among the treatments (Tukey’s test, *p* < 0.05). * Actual limits for extra virgin olive oil (EVOO) category [1,2].

**Table 2:** Fatty acid profile (%) of Buža virgin olive oils obtained from fruits harvested using three different methods: manual harvesting with hand-held combs (B-HH), mechanical harvesting with hand-held shaker rake (B-MH-1), and mechanical harvesting with a self-propelled trunk shaker (B-MH-2).

Fatty Acid (%)	B-HH	B-MH-1	B-MH-2	EVOO *
Myristic (C 14:0)	0.02 ± 0.00	0.02 ± 0.00	0.02 ± 0.01	≤0.03
Palmitic (C 16:0)	14.53 ± 0.31	14.26 ± 0.25	14.50 ± 0.07	7.50–20.00
Palmitoleic (C 16:1)	1.14 ± 0.02	1.15 ± 0.01	1.16 ± 0.01	0.30–3.50
Heptadecanoic (C 17:0)	0.18 ± 0.00	0.18 ± 0.01	0.19 ± 0.00	≤0.40
Heptadecenoic (C 17:1)	0.31 ± 0.00	0.31 ± 0.01	0.30 ± 0.01	≤0.60
Stearic (C 18:0)	2.48 ± 0.02	2.46 ± 0.01	2.49 ± 0.02	0.50–5.00
Oleic (C 18:1)	67.55 ± 0.14	67.74 ± 0.17	67.55 ± 0.04	55.00–85.00
Linoleic (C 18:2)	12.08 ± 0.19	12.16 ± 0.18	12.16 ± 0.05	2.50–21.00
Linolenic (C 18:3)	0.83 ± 0.02	0.84 ± 0.03	0.82 ± 0.01	≤1.00
Arachidic (C 20:0)	0.42 ± 0.01 ^a^	0.41 ± 0.01 ^a^	0.38 ± 0.00 ^b^	≤0.60
Eicosenoic (C 20:1)	0.29 ± 0.01	0.29 ± 0.03	0.26 ± 001	≤0.50
Behenic (C 22:0)	0.12 ± 0.00	0.11 ± 0.00	0.11 ± 0.00	≤0.20
Erucic (C 22:1)	0.00 ± 0.00	0.01 ± 0.00	0.01 ± 0.00	
Lignoceric (C 24:0)	0.05 ± 0.01	0.05 ± 0.00	0.04 ± 0.01	≤0.20
∑SFA	17.80 ± 0.28	17.50 ± 0.25	17.73 ± 0.05	
∑MUFA	69.29 ± 0.13	69.50 ± 0.18	69.28 ± 0.06	
∑PUFA	12.91 ± 0.20	13.00 ± 0.19	12.98 ± 0.05	

Results are expressed as mean values ± standard deviation of three independent repetitions. Mean values labeled with different letters within the same row are statistically different (Tukey’s test, *p* ˂ 0.05). * Actual limits for extra virgin olive oil (EVOO) category [1,2]. SFA—saturated fatty acids, MUFA—monounsaterated fatty acids, PUFA—polyunsaturated fatty acids.

**Table 3:** Concentrations of volatile compounds in Buža virgin olive oils obtained from fruits harvested using three different methods: manual harvesting with hand-held combs (B-HH), mechanical harvesting with hand-held shaker rake (B-MH-1), and mechanical harvesting with a self-propelled trunk shaker (B-MH-2).

Volatile Compound (Odor Threshold *) (mg/kg)	B-HH	B-MH-1	B-MH-2
Octane (0.94)	0.012 ± 0.001	0.010 ± 0.002	0.011 ± 0.002
Ethyl acetate (0.94)	0.022 ± 0.002 ^b^	0.022 ± 0.007 ^b^	0.039 ± 0.005 ^a^
Ethanol (30.0)	0.766 ± 0.210	0.545 ± 0.082	0.759 ± 0.077
Ethyl propanoate (0.10)	0.001 ± 0.000	0.028 ± 0.048	0.033 ± 0.056
Hexanal (0.08)	1.197 ± 0.072 ^a^	1.062 ± 0.027 ^ab^	0.944 ± 0.120 ^b^
3-Methyl-1-butanol (0.10)	0.002 ± 0.000 ^ab^	0.001 ± 0.000 ^b^	0.003 ± 0.000 ^a^
(*E*)-2-Hexenal (0.420)	6.381 ± 0.476 ^ab^	5.801 ± 0.237 ^b^	7.141 ± 0.617 ^a^
(*Z*)-3-Hexenyl acetate (0.2)	0.002 ± 0.001	0.002 ± 0.001	0.003 ± 0.001
(*E*)-2-Heptenal (0.042)	0.043 ± 0.002 ^ab^	0.037 ± 0.001 ^b^	0.051 ± 0.009 ^a^
6-methyl-5-hepten-2-one (1.00)	0.035 ± 0.004 ^ab^	0.025 ± 0.004 ^b^	0.040 ± 0.007 ^a^
1-Hexanol (0.40)	0.132 ± 0.013 ^b^	0.236 ± 0.011 ^a^	0.220 ± 0.009 ^a^
Nonanal (0.15)	0.124 ± 0.018	0.139 ± 0.018	0.112 ± 0.011
1-Octen-3-ol (0.05)	0.005 ± 0.001	0.003 ± 0.000	0.006 ± 0.003
(*E*,*E*)-2,4-Hexadienal	0.200 ± 0.022	0.191 ± 0.016	0.178 ± 0.013
Acetic acid (0.5)	0.236 ± 0.031	0.249 ± 0.013	0.291 ± 0.039
Propanoic acid (0.72)	0.434 ± 0.028	0.391 ± 0.061	0.366 ± 0.019
(*E*)-2-Decenal (0.01)	0.068 ± 0.005	0.067 ± 0.004	0.077 ± 0.008
Pentanoic acid (0.60)	0.011 ± 0.001 ^c^	0.048 ± 0.007 ^a^	0.029 ± 0.002 ^b^

Results are presented as mean values of three technical repetitions ± standard deviations. Different letters in a row represent significant differences among the treatments (Tukey’s test, *p* < 0.05). * Odor threshold values (mg/kg) as reported in the literature [44].

**Table 4:** Concentrations of phenolic compounds of Buža virgin olive oils obtained from fruits harvested using three different methods: manual harvesting with hand-held combs (B-HH), mechanical harvesting with hand-held shaker rake (B-MH-1), and mechanical harvesting with a self-propelled trunk shaker (B-MH-2).

Phenolic Compounds (mg/kg)	B-HH	B-MH-1	B-MH-2
Oleuropein	10.97 ± 2.05	14.71 ± 0.72	11.53 ± 1.93
Oleuropein aglycone, dialdehyde form	12.25 ± 2.57	15.11 ± 1.10	12.78 ± 1.95
Oleuropein aglycone, oxidized aldehyde and hydroxylic form	1.59 ± 0.15	1.69 ± 0.08	1.35 ± 0.27
Oleacein	49.69 ± 8.75	63.24 ± 4.67	48.11 ± 10.68
Oleacein, oxidized form	2.20 ± 0.06 ^b^	2.86 ± 0.16 ^a^	2.26 ± 0.11 ^b^
Oleocanthal	20.62 ± 0.90	22.33 ± 1.20	21.88 ± 0.51
Oleocanthal, oxidized form	6.75 ± 0.81 ^ab^	8.06 ± 0.60 ^a^	5.97 ± 0.34 ^b^
Oleuropein aglycone, aldehyde and hydroxylic form	21.03 ± 1.34	21.72 ± 0.62	21.91 ± 0.66
Ligstroside aglycone, dialdehyde form	28.59 ± 2.28	29.98 ± 1.73	30.49 ± 1.12
Ligstroside aglycone, aldehyde and hydroxylic form	11.71 ± 1.19 ^ab^	9.07 ± 0.34 ^b^	12.93 ± 1.66 ^a^
Ligstroside aglycone, oxidized aldehyde and hydroxylic form	5.28 ± 0.38	5.07 ± 0.51	5.44 ± 0.53
Total secoiridoids	170.67 ± 19.97	193.85 ± 7.10	174.64 ± 16.71
Tyrosol	5.58 ± 0.18	5.74 ± 0.16	5.75 ± 0.38
Hydroxytyrosol	1.04 ± 0.08 ^ab^	1.26 ± 0.11 ^a^	0.89 ± 0.24 ^b^
Hydroxytyrosol acetate	0.15 ± 0.06	0.11 ± 0.05	0.25 ± 0.05
Tyrosol acetate	1.20 ± 0.14 ^ab^	1.54 ± 0.14 ^a^	1.03 ± 0.23 ^b^
Vanillin	4.80 ± 0.48	4.88 ± 0.15	5.36 ± 0.51
Total simple phenols	12.76 ± 0.42	13.54 ± 0.32	13.31 ± 1.20
Vanillic and caffeic acid	1.97 ± 0.12 ^ab^	2.10 ± 0.05 ^a^	1.77 ± 0.05 ^b^
*p*-Coumaric acid	1.99 ± 0.19	2.13 ± 0.10	1.95 ± 0.11
*o*-Coumaric acid	0.25 ± 0.00 ^a^	0.24 ± 0.03 ^a^	0.14 ± 0.02 ^b^
Ferulic acid	0.47 ± 0.07	0.55 ± 0.06	0.55 ± 0.04
Cinnamic acid	0.44 ± 0.10 ^b^	0.50 ± 0.33 ^b^	1.23 ± 0.19 ^a^
Total phenolic acids	5.12 ± 0.23	5.51 ± 0.43	5.63 ± 0.04
Pinoresinol, 1-acetoxy-pinoresinol	31.16 ± 0.68	29.56 ± 0.37	30.15 ± 1.20
Total lignans	31.16 ± 0.68	29.56 ± 0.37	30.15 ± 1.20
Luteolin	6.86 ± 0.03 ^b^	7.38 ± 0.48 ^b^	8.39 ± 0.26 ^a^
Apigenin	1.96 ± 0.05 ^b^	2.05 ± 0.10 ^b^	2.43 ± 0.22 ^a^
Methyl-luteolin	0.51 ± 0.03 ^b^	0.52 ± 0.04 ^ab^	0.59 ± 0.02 ^a^
Total flavonoids	9.33 ± 0.10 ^b^	9.95 ± 0.61 ^b^	11.42 ± 0.48 ^a^
Total phenolic content	229.03 ± 21.30	252.41 ± 7.27	235.15 ± 13.98

Results are presented as mean values of three technical repetitions ± standard deviations. Different letters in a row represent significant differences among the treatments (Tukey’s test, *p* < 0.05).

## Data Availability

The original contributions presented in this study are included in the article. Further inquiries can be directed to the corresponding author.

## Abbreviations

The following abbreviations are used in this manuscript:

B-HH	Manual harvesting using hand-held combs
B-MH-1	Mechanical harvesting using hand-held shaker rake
B-MH-2	Mechanical harvesting by self-propelled trunk shaker
VOO	Virgin olive oil
EVOO	Extra virgin olive oil
PV	Peroxide value
FFA	Free fatty acidity
LOX	Lipoxygenase pathway
FAEE	Fatty acid ethyl esters
MI	Maturity Index
IOC	International Olive Council
FID	Flame ionization detector
HS-SPME	Headspace solid-phase microextraction
GC-FID/MS	Gas chromatography-flame ionization detection and mass spectrometry
DVB/CAR/PDMS	Divinylbenzene/carboxen/polydimethylsiloxane
HPLC-DAD	High-performance liquid chromatography with a diode array detector
3,4-DHPEA-EDA	Decarboxymethyl oleuropein aglycone, dialdehyde form
p-HPEA-EDA	Decarboxymethyl ligstroside aglycone dialdehyde form
ANOVA	One-way analysis of variance
SFA	Saturated fatty acids
MUFA	Monounsaterated fatty acids
PUFA	Polyunsaturated fatty acids
TPC	Total phenolic content
